# Maternity leave and exclusive breastfeeding

**DOI:** 10.11606/S1518-8787.2019053000244

**Published:** 2019-01-18

**Authors:** Karina Abibi Rimes, Maria Inês Couto de Oliveira, Cristiano Siqueira Boccolini

**Affiliations:** I Universidade Federal Fluminense. Instituto de Saúde Coletiva. Programa de Pós-Graduação em Saúde Coletiva. Niterói, RJ, Brasil; II Universidade Federal Fluminense. Instituto de Saúde Coletiva. Departamento de Epidemiologia e Bioestatística. Niterói, RJ, Brasil; III Fundação Oswaldo Cruz. Instituto de Comunicação e Informação Científica e Tecnológica em Saúde. Laboratório de Informação em Saúde. Rio de Janeiro, RJ, Brasil

**Keywords:** Breast Feeding, Parental Leave, Cross-Sectional Studies, Public Health Policy, Maternal and Child Health, Aleitamento Materno, Licença Parental, Estudos Transversais, Políticas Públicas de Saúde, Saúde Materno-Infantil

## Abstract

**OBJECTIVE:**

To analyze the association between maternity leave and exclusive breastfeeding and to estimate the prevalence of exclusive breastfeeding in children under six months of life.

**METHODS:**

Cross-sectional study, with mothers of children under six months of life, attended in primary health care units with Breast Milk Collection Services in the municipality of Rio de Janeiro, Brazil, in 2013 (n = 429). We analyzed characteristics concerning: maternal sociodemographic aspects, household, prenatal care, childbirth, maternal lifestyle, the child, health care, and infant feeding. Adjusted prevalence ratios (APR) were obtained by Poisson regression with robust variance according to hierarchical approach, and we kept in the final model variables that were associated (p ≤ 0.05) with exclusive breastfeeding (outcome).

**RESULTS:**

Among the interviewed mothers, 23.1% were on maternity leave and 17.2% were working. The prevalence of exclusive breastfeeding was 50.1%. The maternal work with maternity leave was associated with higher prevalence of the outcome (APR = 1.91; 95%CI 1.32–2.78), compared with mothers who worked without maternity leave.

**CONCLUSIONS:**

Maternity leave has contributed to the practice of exclusive breastfeeding for children under six months of life, which indicates the importance of this benefit in protecting exclusive breastfeeding for women inserted in the formal labor market.

## RESUMO

**OBJETIVO:**

Analisar a associação entre a licença-maternidade e o aleitamento materno exclusivo e estimar a prevalência de aleitamento materno exclusivo em crianças menores de seis meses de vida.

**MÉTODOS:**

Estudo transversal, com mães de crianças menores de seis meses, assistidas por unidades básicas de saúde com Posto de Recolhimento de Leite Humano Ordenhado no município do Rio de Janeiro, Brasil, em 2013 (n = 429). Foram analisadas características sociodemográficas maternas, domiciliares, da assistência pré-natal, do parto, do estilo de vida materno, da criança, da assistência à saúde e da alimentação infantil. Razões de prevalências ajustadas foram obtidas por regressão de Poisson com variância robusta, segundo abordagem hierarquizada, sendo mantidas no modelo final as variáveis que se associaram (p ≤ 0,05) ao aleitamento materno exclusivo (desfecho).

**RESULTADOS:**

Entre as mães entrevistadas, 23,1% estavam em licença-maternidade e 17,2% estavam trabalhando. A prevalência de aleitamento materno exclusivo em menores de seis meses foi de 50,1%. O trabalho materno com licença-maternidade esteve associado a uma maior prevalência do desfecho (RPa = 1,91; IC95% 1,32–2,78), comparado às mães que trabalhavam sem licença-maternidade.

**CONCLUSÕES:**

A licença-maternidade contribuiu para a prática do aleitamento materno exclusivo em crianças menores de seis meses de vida, o que indica a importância desse benefício na proteção do aleitamento materno exclusivo para as mulheres inseridas no mercado de trabalho formal.

## INTRODUCTION

In 2015, 40.4 million of Brazilian women were inserted in the labor market, which accounted for 42.8% of the population in the country^a^; a reality that can interfere with the practice of exclusive breastfeeding in this population^1^. The association between maternal work and exclusive breastfeeding has been widely investigated in epidemiological studies in Brazil. However, few studies consider the maternity leave^2^.

Worldwide, literature suggests that policies in favor of maternity leave are effective in increasing the practice of exclusive breastfeeding in the first six months of children's life^3^, period recommended by the World Health Organization for exclusive breastfeeding^b^. However, hundreds of millions of working women in the world have no maternity protection yet, or have it inappropriately^3^. In Brazil, the 120-day maternity leave^c^ follows the minimum recommendations of the International Labor Organization of 14 weeks, and its extension to 180 days would meet the broad recommendations of 18 weeks^d^.

Investing in maternity leave can exert positive impacts on women's and children's health and on the economy of the country^3^. By protecting exclusive breastfeeding, there would be decrease in maternal and child morbidity and mortality, increase in the children's intelligence quotient (IQ) and school performance, thus indirectly contributing to alleviating poverty^4^. Our study aimed to analyze the association between maternity leave and exclusive breastfeeding and to estimate the prevalence of exclusive breastfeeding in children under six months of life.

## METHODS

### Study Design and Sample

This is an epidemiological study based on a survey conducted in 2013, entitled “Evaluation of factors associated with breast milk donation by users of primary health care units in Rio de Janeiro City.” The research population was composed of a representative sample of mothers of children under one year of age attended at nine primary health care units of the Municipal Department of Health of Rio de Janeiro, which featured Breast Milk Collection Services in 2013^5^. Units were distributed in five of the ten planning areas of the city^e^, being six Family Clinics and three Municipal Healthcare Centers. Primary Health Care Units with Breast Milk Collection Services were selected because we aimed to analyze breast milk donation and cross-nursing. This research considered the prevalence of cross-nursing as the basis for estimating the sample size (estimated at 50% based on pilot study), which resulted in a sample size of 697 mothers, considering a monthly average of 1,321 children under one year of age attended at the selected primary health care units^5^. The survey was conducted according to the regulatory norms for research involving human beings established by Resolution CNS 466/12 and was approved by the Research Ethics Committee of the Municipal Department of Health of Rio de Janeiro (Opinion 228A/2013, CAAE 49306615.0.0000.5243).

The hypothesis of this study is that maternity leave contributes to the practice of exclusive breastfeeding, which is the outcome of our investigation. Since exclusive breastfeeding is an indicator estimated for children in the first half of their life^b^, were selected mothers of children in this age group (n = 429), of the total of mothers of children under one year old interviewed for the survey. This sample number was enough to detect a prevalence ratio of 2.0, with 80% power of test, and 5% alpha, accounting for a prevalence of 27% of the outcome in the nonexposed group (working mothers with no maternity leave), and 70% in the exposed group (mothers on maternity leave). These parameters were estimated *a posteriori* with data obtained from the research^6^.

### Data Collection

Collection of research data occurred in November and December 2013, by interviews with structured questionnaires. Instruments comprised questions concerning the following characteristics: maternal sociodemographic aspects, household, prenatal care, childbirth, maternal lifestyle, the child, health care, and infant feeding. Data were collected by signing the informed consent form. Questionnaires were previously tested in a pilot study in two primary health care units of the same municipality, which did not participate in the research, in the two months prior to data collection. The six selected interviewers (nurses or nutritionists) were similarly trained in theoretical-practical training programs, participated in the pilot study, and worked under supervision. Data were collected in all shifts of the healthcare services until reaching the sample number provided for each unit.

The outcome of the study was the exclusive breastfeeding, defined as children receiving breast milk only, straight from the breast or milked, or human milk from another source, without the addition of other liquids or solid foods, with the exception of drops of syrup containing vitamins, mineral supplements, oral rehydration salts, or medicines^b^. Thus, children's feeding was verified by closed questions about the consumption of breast milk, water, teas, other liquids, other types of milk, and other foods in the last 24 hours (current status). The World Health Organization recommends the indicator to be based on data from the current status for being a method deemed appropriate for research on food intake whose objective is to describe infant feeding practices in populations^b^.

The “maternity leave,” main focus of our study, was questioned only to mothers who reported having formal employment relationship, in order to know whether they were on leave or had returned to work. Possible confounding factors selected for the analysis of the association between maternity leave and exclusive breastfeeding were organized based on the systematic review by Boccolini et al.^2^ Thus, a hierarchical theoretical model was developed, according to the level of proximity to the outcome, in groups of variables, namely: distal (maternal and household characteristics – model 1), distal intermediate (prenatal care characteristics – model 2), proximal intermediate (childbirth and newborn characteristics – model 3), and proximal (nursing mothers, children, and health care characteristics – model 4), as shown in Figure. In the proposed model, the sex of the baby can be a factor interfering with exclusive breastfeeding, but it is not determined by any of the variables of the previous models. Therefore, it was selected for adjustment in the statistical modeling, but it was considered apart from the theoretical model. This hierarchical approach allows obtaining a more parsimonious statistical model^7^.

**Figure:**
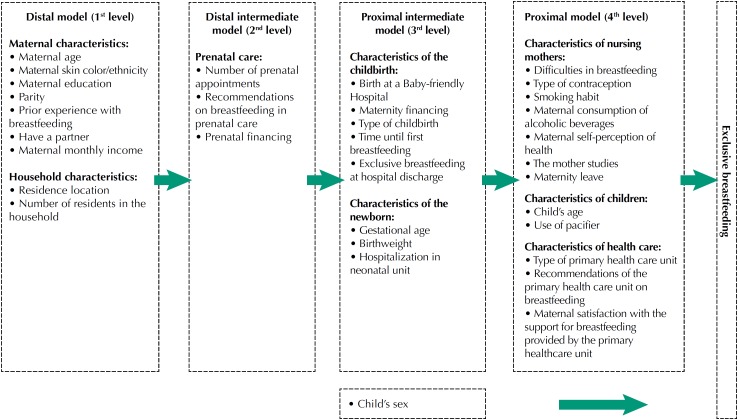
Hierarchical theoretical model of factors associated with exclusive breastfeeding.

The variable “maternal skin color/ethnicity” was self-reported, initially obtained according to the classification of the Brazilian Institute of Geography and Statistics (IBGE)^f^ (mixed race, black, yellow, and indigenous) and subsequently categorized into “white” and “non-white.” “Maternal education” was measured according to the last year of school reported by the mother, and then dichotomized in “less than high school” and “high school or more.” “Maternal monthly income” was obtained in the Brazilian currency, *real* (BRL), and categorized into “no income,” “greater than or equal to one minimum wage,” and “less than one minimum wage.” Mothers were questioned about difficulties while breastfeeding their child such as: “cracked nipple,” “milk stasis,” and “mastitis”; and to the positive answer concerning at least one of the items, we considered “yes.” The “maternal self-perception of health” was obtained when mothers were questioned as for the evaluation of their health as “good,” “fairly good,” or “bad,” and the variable was categorized into positive (“good”) and negative (“fairly good” or “bad”). Concerning the guideline of the primary health care unit on breastfeeding, we considered “received at least one recommendation” for positive responses of at least one of the following five questions. Did someone at this unit: “explain that when babies are born they should be exclusively breastfed?”; “show how to put the baby on the breast to suckle?”; “explain that babies should be breastfed whenever they want?”; “explain how to express breast milk with your hands (or with a pump) if necessary?”; and “advise that babies should not be bottle fed?”. As for the variable “maternal satisfaction with breastfeeding support provided at the primary health care unit,” mothers were questioned on whether the unit was helping them or had helped them to breastfeed; answers “yes” or “kind of” were categorized into “satisfied,” and “no,” into “unsatisfied.”

### Data Analysis

For data entry the EpiInfo 2000 program was used, and data analysis was performed with the SPSS17 program. Distribution of variables of exposure and outcome was known by univariate analysis. Subsequently, the bivariate analysis was conducted between each variable of exposure and the outcome, exclusive breastfeeding. Pearson's Chi-square test was used for obtaining crude prevalence ratios (CPR) with their respective 95% confidence intervals (95%CI). Variables with p ≤ 0.20 in the bivariate analysis were included in the multiple analysis. As for multiple analysis, adjusted prevalence ratios (APR) were obtained by the Poisson regression model with robust variance^8^. Exposure variables were adjusted according to the time proximity with the outcome^7^ (Figure).

Thus, we initially included in the model maternal and household characteristics (first level) and those associated with exclusive breastfeeding at a significance level less than or equal to 5% (p ≤ 0.05) were kept in the modeling. Then, we included characteristics concerning prenatal care (second level), which were adjusted by the first level exposure variables that remained in the model. The same occurred with the characteristics of the third level (related to childbirth and the newborn), which were adjusted by the variables of the first and second levels that remained in the modeling. Finally, we added the characteristics of the fourth level (characteristics of nursing mothers, children, and health care), which were adjusted by the variables of the three previous levels maintained in the model.

## RESULTS

The prevalence of exclusive breastfeeding accounted for 50.1%. Most mothers claimed to have non-white skin color/ethnicity, have a partner, and reside in a low-income community (Table 1). Most mothers were followed up by at least six prenatal appointments, only a quarter of the children were born at a Baby-friendly Hospital, more than half of the childbirths were normal, and three quarters of children were in exclusive breastfeeding at hospital discharge (Table 2). Less than half of the mothers reported having formal employment relationship, of which most were on maternity leave at the time of the interview (Table 3).

**Table 1:** Prevalence and crude prevalence ratio of exclusive breastfeeding among children under six months of life according to distal characteristics. Rio de Janeiro, state of Rio de Janeiro, Brazil, 2013. (n = 429)

Variable		n	%	EBF (%)	CPR	p
Maternal characteristics						
Maternal age (years)						
	20 to 45	331	77.2	53.2	1	
	13 to 19	98	22.8	39.8	0.748	0.031
Maternal skin color/ethnicity						
	White	101	23.5	59.4	1	
	Non-white	328	76.5	47.3	0.795	0.023
Maternal education						
	High school or more	162	37.8	56.2	1	
	Less than high school	267	62.2	46.4	0.827	0.047
Parity						
	Multiparous	210	49.0	52.4	1	
	Primipara	219	51.0	47.9	0.915	0.359
Prior experience with breastfeeding						
	Breastfed for ≥ 6 months	152	35.4	55.9	1	
	Breastfed for < 6 months	277	64.6	46.9	0.839	0.069
Have a partner						
	Yes	375	87.4	52.8	1	
	No	54	12.6	31.5	0.596	0.012
Maternal monthly income						
	No income	164	38.3	53.7	1	
	≥ 1 minimum wage	144	33.6	52.8	0.984	0.877
	< 1 minimum wage	120	28.0	42.5	0.792	0.070
Household characteristics						
Residence location						
	Neighborhood	162	37.8	51.2	1	
	Low-income community	267	62.2	49.4	0.965	0.717
Number of residents in the household						
	Up to 4	270	62.9	49.3	1	
	5 or over	159	37.1	51.6	1.047	0.642

EBF: exclusive breastfeeding; CPR: crude prevalence ratio

**Table 2:** Prevalence and crude prevalence ratio of exclusive breastfeeding among children under six months of life according to intermediate characteristics. Rio de Janeiro, state of Rio de Janeiro, Brazil, 2013. (n = 429)

Variable		n	%	EBF (%)	CPR	p
Distal intermediate characteristics						
Number of prenatal appointments						
	≥ 6	379	88.3	52.5	1	
	< 6	50	11.7	32.0	0.609	0.019
Recommendations on breastfeeding in prenatal care						
	Yes	338	78.8	50.3	1	
	No	91	21.2	50.1	0.983	0.887
Prenatal financing						
	Brazilian Unified Health System	393	91.6	50.4	1	
	Other	32	7.5	50.0	0.992	0.967
	No prenatal care	4	0.9	25.0	0.496	0.419
Proximal intermediate characteristics						
Birth at a Baby-friendly Hospital						
	Yes	118	27.6	55.1	1	
	No	309	72.4	48.2	0.875	0.192
Maternity financing						
	Brazilian Unified Health System	386	86.2	49.5	1	
	Other	59	13.8	54.2	1.097	0.480
Type of childbirth						
	Normal	247	57.6	48.2	1	
	Cesarean section	182	42.4	52.8	1.095	0.347
Time until first breastfeeding						
	In the first hour of life	247	57.6	52.2	1	
	After the first hour of life	182	42.4	47.2	0.905	0.313
Exclusive breastfeeding at hospital discharge						
	Yes	336	78.3	53.0	1	
	No	93	21.7	39.8	0.751	0.037
Gestational age						
	Term	388	90.4	51.0	1	
	Preterm	41	9.6	41.5	0.813	0.280
Birthweight						
	≥ 2.500 g	403	93.9	50.4	1	
	< 2.500 g	26	6.1	46.2	0.916	0.688
Hospitalization in neonatal unit						
	No	355	82.8	50.1	1	
	Yes	74	17.2	50.0	0.997	0.982
Child's sex						
	Female	197	45.9	50.8	1	
	Male	232	54.1	49.6	0.977	0.805

EBF: exclusive breastfeeding; CPR: crude prevalence ratio

**Table 3:** Prevalence and crude prevalence ratio of exclusive breastfeeding among children under six months of life according to proximal characteristics. Rio de Janeiro, state of Rio de Janeiro, Brazil, 2013. (n = 429)

Variable		n	%	EBF (%)	CPR	p
Characteristics of nursing mothers						
Difficulties in breastfeeding						
	No	187	43.8	50.3	1	
	Yes	240	56.2	50.0	0.995	0.956
Type of contraception						
	Methods without estrogen	387	90.4	52.4	1	
	Combined pill	41	9.6	26.8	0.511	0.011
Smoking habit						
	No	376	87.6	52.4	1	
	Yes	53	12.4	34.0	0.648	0.028
Maternal consumption of alcoholic beverages					
	No	379	88.3	53.0	1	
	Yes	50	11.7	28.0	0.528	0.006
Maternal self-perception of health						
	Positive	363	84.6	52.6	1	
	Negative	66	15.4	36.4	0.691	0.030
Characteristics of children						
Child's age (months)						
	0 to 1	110	25.6	75.4	1	
	2 to 3	176	41.0	48.9	0.426	< 0.001
	4 to 5	143	33.3	32.2	0.648	< 0.001
Use of pacifier						
	No	236	55.1	58.5	1	
	Yes	192	44.9	40.1	0.686	< 0.001
Characteristics of health care						
Type of primary health care unit						
	Family Clinic	241	56.2	52.3	1	
	Municipal Healthcare Center	188	43.8	47.3	0.905	0.313
Recommendations of the primary health care unit on breastfeeding						
	No recommendations	26	6.1	30.8	1	
	Received at least one recommendation	403	93.9	51.4	1.669	0.086
Maternal satisfaction with breastfeeding support provided by the primary health care unit						
	Satisfied	336	78.3	52.1	1	
	Unsatisfied	93	21.7	43.0	0.826	0.142
The mother studies						
	Yes	15	3.5	26.7	1	
	Does not study	384	89.9	50.0	1.875	0.145
	Student leave	28	6.6	67.9	2.545	0.037
Maternity leave						
	No	74	17.2	27.0	1	
	Without paid work or self-employed	256	59.7	49.2	1.821	0.003
	Yes	99	23.1	69.7	2.579	< 0.001

EBF: exclusive breastfeeding; CPR: crude prevalence ratio

In the bivariate analysis, we observed that the following characteristics were associated with lower prevalence of exclusive breastfeeding (p ≤ 0.20), namely: distal – adolescence, non-white maternal skin color/ethnicity, maternal education less than high school, prior experience with breastfeeding for less than six months, having no partner, and maternal monthly income less than one minimum wage (Table 1); distal intermediate – less than six prenatal appointments; proximal intermediate – childbirth at a non-baby-friendly hospital and children not in exclusive breastfeeding at hospital discharge (Table 2); and proximal – using combined contraceptive pill, smoking habit, maternal consumption of alcoholic beverages, negative maternal self-perception of health, children in the second or third bimester of life, use of pacifier, mothers not oriented by the primary health care unit on breastfeeding, mothers unsatisfied with the support provided by the primary health care unit on breastfeeding, mothers studying and working with formal employment relationship without maternity leave at the time of the interview (Table 3).

In the multiple hierarchical modelling, the following variables were associated with lower prevalence of the outcome (p ≤ 0.05): distal – maternal non-white skin color/ethnicity and having no partner; intermediate – less than six prenatal appointments; and proximal – maternal consumption of alcoholic beverages, increasing age of the child in months, and use of pacifier. The proximal variable category, mothers on maternity leave, was associated with greater prevalence of the outcome (Table 4).

**Table 4:** Adjusted prevalence ratio of exclusive breastfeeding among children under six months of life. Rio de Janeiro, state of Rio de Janeiro, Brazil, 2013. (n = 429)

Variable		APR	95%CI	p
Distal characteristics				
Maternal skin color/ethnicity				
	White	1		
	Non-white	0.807	0.663–0.982	0.032
Have a partner				
	Yes	1		
	No	0.604	0.603–0.905	0.014
Intermediate characteristic				
Number of prenatal appointments				
	≥ 6	1		
	< 6	0.624	0.413–0.941	0.025
Proximal characteristics				
Maternal consumption of alcoholic beverages				
	No	1		
	Yes	0.601	0.398–0.908	0.016
Child's age				
	Increasing age in months	0.692	0.615–0.779	< 0.001
Use of pacifier				
	No	1		
	Yes	0.672	0.560–0.807	< 0.001
Maternity leave				
	No	1		
	Without paid work or self-employed	1.550	1.079–2.226	0.018
	Yes	1.913	1.323–2.766	0.001

APR: adjusted prevalence ratio

## DISCUSSION

Exclusive breastfeeding accounted for half of the children, a situation considered good according to the parameters of the World Health Organization^g^. We found a prevalence of exclusive breastfeeding greater than 33.3% observed in 2006 in the same municipality. This may be due to the tendency of the increasing practice of exclusive breastfeeding observed in a time-series study^9^, or because of the difference of the studied population, since we selected only primary health care units which had Breast Milk Collection Services, where mothers are expected to receive more support and recommendation to breastfeed. Nevertheless, the practice of exclusive breastfeeding was below the six months recommended by the World Health Organization^b^.

In our study, less than a quarter of the mothers were on maternity leave at the time of the interview. Such mothers showed a prevalence of exclusive breastfeeding 91% higher than those working without maternity leave, which shows the importance of this protection for the practice of exclusive breastfeeding. Maternity leave allows breastfeeding mothers to keep a safe source of income in a period in which they need to be close to their children, enabling greater dedication to them, and thus it is deemed a facilitator of exclusive breastfeeding^10^. Mothers on maternity leave had higher prevalence of exclusive breastfeeding even when compared with mothers without paid work, indicating that maternal work does not seem to hinder exclusive breastfeeding, but actually the lack of maternity leave.

The historical background of social exclusion and oppression of women in the labor market has placed women in disadvantage when compared with men, which, in addition to the double burden (household and paid works), has been overloading women, generating even greater prevalence of occupational diseases in this population^11^.

However, female participation in the labor market increases every year since the 1970s. The double burden is becoming more frequent and women have to manage the care provided to their children with their formal workload. When they have no support network, such fact may negatively interfere in the quality of the care provided to their children, especially those concerning their feeding^12^. According to *Pesquisas Nacionais por Amostra de Domicílio* (PNAD – National Household Sample Survey), from 2001 to 2009, more women were inserted in the informal labor market in this period, reinforcing gender inequality^13^. In 2016, 42.2% of women employed in Brazil were inserted in the informal labor market^a^.

This unfavorable scenario to working women inserted in the informal labor market may explain the greater prevalence of exclusive breastfeeding found among those protected by maternity leave, which is supported by our findings. It is assumed that the mothers who can benefit from the maternity leave, for a predefined and temporary period, seek to make the most of this time to be close to their children, exclusively breastfeeding them.

In countries where maternity leave is much shorter than in Brazil, such as in the United States of America, the chance of women to not initiate breastfeeding and early interrupting it is high^14^, whereas in Nordic and Eastern European countries, where maternity leave is more extensive, women breastfeed for long periods^15^.

The Brazilian legislation, with the promulgation of the Constitution of 1988, prolonged maternity leave from 84 to 120 days^c^. Despite such progress, maternity leave does not reach 180 days yet, which are recommended by the healthcare field and includes only women who are inserted in the formal labor market. In 2010, the National Congress approved the *Programa Empresa Cidadã* (Citizen Company Program), prolonging maternity leave to 180 days^h^. However, since it is a voluntary concession on the part of companies, its adherence is relatively low. In 2016, only 19,641 companies had joined the Program, which corresponded to less than 10% of companies^i^.

By the same program, the paternity leave can also be extended for 15 days more. It is noteworthy that this also occurs voluntarily on the part of the companies. The presence of the partner is important for the exclusive breastfeeding, becoming a support for breastfeeding women^16^ ^,^ ^17^.

Hence, we perceive that comprehensive initiatives considering maternity leave for all Brazilian workers, and in accordance with the recommended six-month period, are still non-existent in the country. As alternatives for mothers returning to formal work in the first six months of children's life, the right to two extra breaks to breastfeed, half an hour each^j^, and the availability of rooms for breastfeeding in some companies^k^, where mothers have a space for milking and storing breast milk, may contribute to the maintenance of exclusive breastfeeding in this period and for a longer breastfeeding duration^18^.

Having a family support network and organizing themselves to continue to breastfeed their babies during the workday are aspects mentioned for the success of breastfeeding. According to a qualitative study on the state of Paraíba, Brazil, mothers who were informal workers create alternatives to continue breastfeeding such as taking the baby to the workplace, and milking and storing their milk, in order to maintain the baby's health^19^. Networks of support for pregnant women and nursing mothers at primary health care also play an important role in maintaining exclusive breastfeeding. Both *Rede Amamenta e Alimenta Brasil* (Brazilian Breastfeeding and Feeding Network) which proposes a flowchart concerning health care provided for the mother or the baby, and promotes breastfeeding^20^, and the *Iniciativa Unidade Básica Amiga da Amamentação* (Breastfeeding-Friendly Primary Care Initiative), which advocates Ten Steps to Successful Breastfeeding, such as groups of mothers to exchange experiences and for mutual support, have been effective in extending the duration of exclusive breastfeeding^21^.

Studies on the association between exclusive breastfeeding and maternal work are more frequent than those about the association with maternity leave. According to a systematic review on factors associated with exclusive breastfeeding in Brazil^2^, only four out of 20 studies that analyzed the variable “working mother” considered the maternity leave. Among these, only one showed statistically significant association: Queluz et al.^10^, in a survey conducted in the city of Serrana, state of São Paulo, Brazil, in 2009, noted that the chance of interrupting exclusive breastfeeding was three times higher among mothers who were not on maternity leave, compared with those who benefit from it.

In addition to maternity leave, we found other variables statistically significant that were associated with exclusive breastfeeding. Non-white maternal skin color/ethnicity, having no partner, less than six prenatal appointments, and children's use of pacifier were negatively associated with exclusive breastfeeding, similarly to other epidemiological studies^16^ ^,^ ^17^ ^,^ ^22^ ^-^ ^25^.

According to popular culture, the consumption of alcoholic beverages is often indicated for lactating women for bringing a feeling of relaxation and supposedly increasing milk production. However, researchers observed that consuming alcoholic beverages raises the level of hormones against milk production^26^, thus decreasing it^27^, which may explain the negative association between alcoholic beverages consumption and exclusive breastfeeding we found in this study.

Although temporality between variables of exposure and the outcome cannot always be established in cross-sectional studies, we presume that maternity leave promotes exclusive breastfeeding, and not the opposite. Another limitation worth mentioning is that we could not differentiate mothers who benefited from the regular period of 120 days of maternity leave from those who benefited for 180 days on the part of citizen companies and for periods longer than 120 days provided in public services^l^.

We conclude that maternity leave has contributed to the practice of exclusive breastfeeding in children under six months of life, which indicates the importance of this benefit in protecting exclusive breastfeeding for women inserted in the formal labor market. We expect the results to strengthen the existing initiatives in the country to extend maternity leave to six months, facilitating the practice of exclusive breastfeeding for the same period. Thus, health care and employment sectors can work together concerning the protection of breastfeeding for working women. Moreover, when highlighting the importance of maternity leave for the group of formal workers, we expect there may be advances in this sense, to comprehensively contemplate all working women. Finally, for a better understanding of the theme, we recommend to carry out qualitative assessments and studies on the impact of the extension of maternity leave (from the current 120 days in Brazil to 180 days) on exclusive breastfeeding, since it would help to create evidence for decision-making on the national scope in order to extend the maternity leave.

## Footnotes

a Instituto Brasileiro de Geografia e Estatística, Coordenação de População e Indicadores Sociais. Síntese de indicadores sociais: uma análise das condições de vida da população brasileira: 2016. Rio de Janeiro: IBGE; 2016 [cited 2017 Oct 20]. (Estudos e pesquisas. Informação demográfica e socioeconômica, 36). Available from: https://biblioteca.ibge.gov.br/visualizacao/livros/liv98965.pdf

b World Health Organization, Department of Child and Adolescent Health and Development. Indicators for assessing infant and young child feeding practices. Part 1 -definitions: conclusions of consensus meeting held 6-8 November 2007 in Washington, DC, USA. Geneva: WHO; 2008 [cited 2017 Oct 20]. Available from: http://apps.who.int/iris/bitstream/handle/10665/43895/9789241596664_eng.pdf;jsessionid=BD45938FF52B5781AD2079CFE1FF9CAC?sequence=1

c Brasil. Constituição (1988). Constituição da República Federativa do Brasil. Brasília (DF): Senado Federal; 1988.

d Addari L, Cassirer N, Gilchrist K. Maternity and paternity at work: law and practice across the world. Geneva: ILO; 2014 [cited 2017 Oct 20]. Available from: http://www.ilo.org/wcmsp5/groups/public/---dgreports/---dcomm/---publ/documents/publication/wcms_242615.pdf

e Câmara Municipal do Rio de Janeiro. Lei complementar N° 111, de 1° de fevereiro de 2011. Dispõe sobre a Política Urbana e Ambiental do Município, institui o Plano Diretor de Desenvolvimento Urbano Sustentável do Município do Rio de Janeiro e dá outras providências. Diario Oficial da Câmara. 29 mar 2011. [cited 2017 Oct 20]. Available from: http://www.rio.rj.gov.br/dlstatic/10112/4600307/4117400/lei_compl_111.pdf

f Instituto Brasileiro de Geografia e Estatística. Pesquisa das características étnico-raciais da população – PCERP. Rio de Janeiro: IBGE; 2012.

g World Health Organization. Infant and young child feeding: a tool for assessing national practices, policies and programmes. Geneva: WHO; 2003 [cited 2017 Oct 20]. Available from: http://apps.who.int/iris/handle/10665/42794

h Brasil. Lei N° 11.770, de 9 de setembro de 2008. Cria o Programa Empresa Cidadã, destinado à prorrogação da licença-maternidade mediante concessão de incentivo fiscal, e altera a Lei n.° 8.212, de 24 de julho de 1991. *Diario Oficial Uniao*. 10 set 2008; Seção 1:1.

i Ministério da Fazenda (BR), Receita Federal. Empresa Cidadã. Brasília (DF); 2016 [cited 2017 Oct 20]. Available from: http://idg.receita.fazenda.gov.br/orientacao/tributaria/isencoes/programa-empresa-cidada/empresa_cidada_relacao_adesoes_01-07-2016.xls/view

j Brasil. Decreto-Lei N° 452, de 1° de maio de 1943. Aprova a Consolidação das Leis do Trabalho. Diario Oficial Uniao. 9 ago 1943 [cited 2017 Oct 20]. Available from: http://www.planalto.gov.br/ccivil_03/decreto-lei/Del5452.htm

k Ministério da Saúde (BR), Agência Nacional de Vigilância Sanitária. Portaria N° 193, de 23 de fevereiro de 2010. Discorre sobre a instalação de salas de apoio à amamentação em empresas públicas ou privadas, e a fiscalização desses ambientes pelas vigilâncias sanitárias locais. Diario Oficial Uniao. 24 fev 2010; Seção 1:98.

1 Brasil. Emenda Constitucional N° 41, de 19 de dezembro de 2003. Modifica os arts. 37, 40, 42, 48, 96, 149 e 201 da Constituição Federal, revoga o inciso IX do § 3 do art. 142 da Constituição Federal e dispositivos da Emenda Constitucional n° 20, de 15 de dezembro de 1998, e dá outras providências. Diario Oficial Uniao. 31 dez 2003 [cited 2017 Oct 20]. Available from: http://www.planalto.gov.br/ccivil_03/constituicao/emendas/emc/emc41.htm
